# Comparative Analysis of Spine and Pelvis Impairment Rating Using the *AMA Guides* Sixth Edition 2024 vs. 2008: Impact on Stakeholders

**DOI:** 10.3390/jcm14061919

**Published:** 2025-03-12

**Authors:** J. Mark Melhorn, Barry Gelinas, Douglas W. Martin, Kurt T. Hegmann, Matthew S. Thiese

**Affiliations:** 1 Department of Orthopedics, School of Medicine, University of Kansas, Wichita, KS 67214, USA; 2 International Academy of Independent Medical Evaluators, Vancouver, WA 98683, USA; barry@bgelinasmddc.com; 3 CNOS Occupational Medicine, Dakota Dunes, SD 57049, USA; douglaswaynemartin7@gmail.com; 4 Rocky Mountain Center for Occupational and Environmental Health, University of Utah, Ogden, UT 84403, USA; kurt.hegmann@hsc.utah.edu (K.T.H.); matt.thiese@hsc.utah.edu (M.S.T.)

**Keywords:** AMA, impairment, rating, spine, pelvis, comparative, analysis, stakeholders

## Abstract

**Background/Objectives**: We aimed to assess the impact of updates in the *2024 AMA Guides to the Evaluation of Permanent Impairment*, Sixth Edition, on spine and pelvis impairment ratings compared to the 2008 Sixth Edition and to investigate potential correlations with legislative, judicial, and economic factors. **Methods**: Nineteen clinical vignettes focused on spine and pelvis conditions were analyzed by two expert evaluators, comparing the impairment ratings derived from both editions. **Results**: Following the spine and pelvis impairment rating procedures from each edition, the 2024 impairment values showed a high correlation and statistical equivalence to those derived from the 2008 methods. **Conclusions**: The *AMA Guides* Sixth Edition 2024 update for spine and pelvis impairments offers enhanced diagnosis-based impairment tables and more transparent processes, improving efficiency while preserving the accuracy, validity, and reliability of previous editions.

## 1. Introduction

The *AMA Guides to the Evaluation of Permanent Impairment (AMA Guides)* are the recognized gold standard for impairment assessment, widely utilized by medical professionals and compensation boards both in the United States and internationally [1,2,3,4,5]. Despite their broad application, each edition of the *AMA Guides* has encountered the criticisms listed below [6,7,8,9,10,11].

- Insufficient inclusion of input from diverse stakeholders, including advocates, attorneys, policymakers, legislators, and unions, during the development process;
- Significant training requirements and a steep learning curve for evaluators;
- Limited testing to evaluate the reliability, reproducibility, precision, and validity of grade modifiers;
- Challenges related to the complexity of conducting impairment evaluations;
- Higher impairment ratings assigned for subjective measures of discomfort and functional limitations;
- Failure to align with the World Health Organization’s International Classification of Functioning, Disability, and Health (ICF) model.

To address these concerns, the *AMA Guides* Editorial Panel (Guides Panel), established in June 2019, formed a musculoskeletal subcommittee in August 2022, which undertook updates using the RAND/UCLA modified Delphi Appropriateness Method and public feedback (Table 1).

We acknowledge that this article shares similarities in study design with a previously published article on upper limb impairments in JOEM, Volume 66, Number 10, October 2024. This similarity plays a crucial role in confirming that a standardized and consistent analytical approach was employed across various body regions, ensuring reliability and uniformity in the assessment methodology. However, it is essential to note that the Specific Individual Elements (SIEs) for assessing impairment in the spine and pelvis differ significantly from those used for the upper or lower limbs. These differences stem from the unique anatomy, function, clinical findings, and diagnostic criteria associated with spine and pelvis impairments, necessitating a distinct comparative analysis.

This article evaluates the consistency of impairment ratings for spine and pelvis impairment values between the 2008 and 2024 editions of the *AMA Guides*, Sixth Edition. Consistency between these editions would facilitate a smooth transition to the 2024 methodology, enhancing clarity and reliability while minimizing disruptions and economic implications for stakeholders, as well as judicial and legislative processes, while ensuring fairness, accuracy, and applicability across all body systems. Conversely, significant discrepancies could pose challenges for judicial and legislative decision-making, potentially impacting policy development and stakeholder confidence.

## 2. Materials and Methods

This comparative, cross-sectional quality improvement study evaluated impairment ratings through vignettes (examples or scenarios). As it did not involve patient participation, the institutional review board at RedCap Kansas University Medical Center classified it as a quality improvement study, exempt from IRB approval. We followed the relevant recommendations outlined in Strengthening the Reporting of Observational Studies in Epidemiology Statement [12] in the design, analysis, and reporting of this study.

The vignettes for this study are drawn from the *AMA Guides* Sixth Edition 2008 and include 19 spine and pelvis Diagnostic-Based Impairment (DBI) cases. The 2008 vignettes were designed to improve evaluators’ accuracy and consistency by offering step-by-step guidance for specific diagnoses with the assumption that the impairment values were correctly obtained.

The analysis was conducted by two experienced physician members of the Guides Panel, who together have 50 years of expertise in musculoskeletal impairment evaluations. They assessed the 19 vignettes using both the 2008 and 2024 methodologies, relying exclusively on clinical history, physical examination findings, and clinical study data from the vignettes, without access to predetermined impairment values.

Initially, the evaluators applied the 2008 methodology to each vignette, producing impairment values consistent with those published in the *AMA Guides* Sixth Edition 2008, with one exception: example 17-3 should be rated at 10% whole person impairment (WPI) rather than the 11% listed. This situation highlights the complexity embedded in the Sixth Edition 2008. While the default value for grade C is 11% WPI, the application of grade modifiers and a net adjustment of -1 indicated that the correct rating should have been 10% WPI, aligning with grade B, which is the correct reference value used.

The same vignettes were then reassessed using the updated 2024 methodology, featuring new evaluation steps and enhanced DBI tables. Eight weeks later, the evaluators repeated the process for both the 2008 and 2024 editions, with the vignettes presented in a different random order for each evaluator during the second round of assessments.

Eight weeks later, the evaluators repeated the analysis, reviewing the vignettes in a newly randomized order to assess the consistency of the findings. This approach allowed for a systematic evaluation of how the updated 2024 *AMA Guides* methodology influenced WPI ratings compared to the 2008 methodology.

The primary outcome measures in this analysis were the spine and pelvis whole person impairment (WPI) ratings, which represent an individual’s overall functional impairment as a percentage based on the *AMA Guides’* standardized scale [13]. Two experienced physician evaluators independently applied the 2008 methodology to each of the 19 vignettes, first applying the 2008 methodology to document WPI ratings. The same vignettes were subsequently re-evaluated using the updated 2024 methodology, with WPI ratings recorded again. A comparative analysis evaluated the differences in impairment ratings for each vignette between the 2008 and 2024 methodologies.

The Spearman correlation statistic was used to measure the relationship between the impairment scores from both methodologies. To determine the significance of differences in median impairment ratings, Wilcoxon’s signed-rank test, a nonparametric statistical method, was applied, and *p*-values were calculated for these differences. To evaluate the agreement between two raters measuring a continuous outcome, we calculated the Concordance Correlation Coefficient (CCC). The CCC assesses the degree to which pairs of observations fall on the 45-degree line of perfect concordance, incorporating both precision (correlation) and accuracy (closeness of means). The means, standard deviations, and Pearson’s correlation coefficient were computed for each rater. The CCC was then calculated using these values. The CCC ranges from −1–1−1 to 111, where 111 indicates perfect concordance, 000 indicates no agreement, and −1–1−1 indicates perfect discordance. The CCC combines precision (correlation) and accuracy (mean differences), unlike Spearman’s correlation statistic alone. We also performed a two one-sided test (TOST) for equivalence between the 2008 and 2024 ratings.

## 3. Results

### 3.1. Impairment Rating

For all 19 spine and pelvis vignettes, the final impairment values remained consistent for each evaluator in both rounds of the 2024 assessment. This consistency yielded a high Spearman correlation coefficient of 0.9942 (*p* < 0.0001), reflecting a strong relationship. This statistical measure quantifies the degree of correlation between impairment ratings. A high correlation indicates that the evaluators arrived at similar or identical impairment values for most vignettes across both rounds, emphasizing the reliability, consistency, and reproducibility of the 2024 updated methodology when applied to the same clinical dataset.

This finding indicates that the updated *AMA Guides* 2024 methodology can be applied with strong consistency and minimal variability among evaluators for spine and pelvis impairment ratings. The perfect alignment between the two rounds of evaluation further enhances confidence in the reliability and reproducibility of the impairment ratings using the 2024 methodology.

### 3.2. Comparison of 2008 and 2024 Impairment Ratings

Table 2 displays the impairment rating values assigned by the evaluators based on both the 2024 and 2008 *AMA Guides* methodologies.

For the spine and pelvis impairment values across the 19 vignettes, the following is true:

- Twelve out of nineteen vignettes (63%) had the same impairment values between the two methods.

For the remaining seven vignettes, the following are true:

- Five vignettes had a slightly higher impairment value using the 2024 method;
- Two vignettes had a slightly lower impairment value using the 2024 method.

The two impairment ratings were highly correlated, with a Spearman correlation coefficient of 0.9942. The combined average spine and pelvis impairment value across all 19 vignettes was 9.79 for the 2024 method and 10.11 for the 2008 method. This represents a nonsignificant 0.32 difference in the average impairment value when transitioning from the 2008 to the 2024 methodology (*p* = 0.9298).

The Concordance Correlation Coefficient (CCC) was calculated to assess the agreement between the two raters for the measured outcome. The CCC value was 0.99007, indicating excellent agreement between the two raters.

The high CCC reflects strong concordance, with minimal variability between the raters’ measurements (precision) and a small difference in their mean values (accuracy). The mean and standard deviation for the 2008 ratings were 9.79 and 8.81, respectively, while for 2024, the ratings were 10.11 and 9.45, respectively. The Pearson correlation coefficient (ρ\rhoρ) was 0.993, further supporting the high level of agreement.

This result suggests that the two raters provided highly consistent and reliable measurements, validating the use of their ratings in subsequent analyses.

The potential impact of floor effects on the agreement analyses was considered. Floor effects occur when a substantial portion of the data cluster at the lower bound of the measurement scale, which can artificially inflate measures of agreement like the Concordance Correlation Coefficient (CCC) by constraining variability and masking true disagreement. In this study, if a significant number of observations were near the minimum possible value for one or both raters, it could have led to an overestimation of agreement due to reduced differences between measurements.

To address this, we visualized the data distributions for each rater to assess clustering at the lower bound. While no substantial floor effects were observed, it is important to acknowledge that their presence could bias the estimation of the CCC by limiting variability and influencing the correlation component. Future studies may consider alternative agreement metrics, such as weighted kappa or nonparametric methods, to account for potential data distribution issues. Additionally, exploring interventions to improve measurement sensitivity at the lower end of the scale could mitigate these potential effects.

A test for equivalence assesses the statistical equivalence between two measures. The two one-sided test, assessing equivalence between the 2008 and 2024 impairment ratings, indicates that the impairment ratings from the two methodologies are equivalent and statistically interchangeable.

## 4. Discussion

This comparative analysis of impairment values for specific spine and pelvis conditions provides key insights into the broader impact of methodological updates in the 2024 edition of the *AMA Guides* compared to the 2008 edition. The high consistency in impairment ratings, with only minor variations, suggests that the 2024 updates preserve continuity in the evaluation process while enhancing clarity and precision. These findings indicate that transitioning from the 2008 to the 2024 edition is unlikely to significantly affect impairment ratings in clinical practice, based on the vignettes reviewed in this study and prior assessments of upper limb conditions [14,15]. Additionally, these results suggest that the 2024 edition may help alleviate stress and dissatisfaction previously noted in the workers’ compensation system with earlier editions [7,11,16,17,18,19].

The 2024 updates, created in response to stakeholder requests, specifically focus on the three musculoskeletal chapters covering the upper limb, lower limb, and spine and pelvis. A key enhancement is the improved DBI tables, which allow for the inclusion of multiple diagnostic rows and their corresponding impairment values. This expanded structure provides evaluators with a broader range of options, facilitating more comprehensive and precise assessments while maintaining improved consistency, reliability, and reproducibility (both interrater and intrarater agreement) [14]. The revised methodology promotes transparency and simplicity, which is expected to lead to more consistent impairment evaluations among evaluators and across various jurisdictions.

A key finding of this study was the demonstrated accuracy, consistency, and reproducibility of the 2024 methodology, evidenced by the high rater agreement between the two physician evaluators. This agreement, measured by the Concordance Correlation Coefficient (CCC), was observed in both the 2008 and 2024 editions. The median WPI ratings showed minimal differences between the two editions, with most vignettes yielding identical impairment values. Furthermore, statistical equivalence testing confirmed that ratings from the 2008 and 2024 editions are interchangeable, reinforcing confidence that the 2024 updates will not adversely impact individuals with impairments or economic outcomes.

The *AMA Guides* remain the international gold standard for objective, evidence-based impairment evaluation, integrating the latest clinical and scientific knowledge [4,7,20]. The 2024 edition builds on the strengths of previous versions, delivering fair and equitable impairment values grounded in scientific evidence. This study demonstrates that the 2024 methodology improves usability, consistency, reliability, and reproducibility without significantly altering impairment values for spine and pelvis conditions.

Impairment determination is a multi-step process, where transparency and reproducibility are critical for judicial and adjudicative accuracy. This comparative analysis confirms that the 2024 updates effectively achieve these objectives. While the anticipated impact on legal and workers’ compensation systems is minimal, the benefits for evaluators—including enhanced clarity, ease of use, and improved consistency—are substantial.

This study has several limitations, mainly its dependence on observational data from clinical vignettes rather than in-person evaluations. Standardized hypothetical cases derived from the *AMA Guides* 2008 vignettes were utilized to maintain a consistent clinical dataset. Although this method enables controlled comparisons, it may not fully reflect the complexities of live patient assessments. However, by minimizing real-world variability, it reduces the likelihood of incomplete evaluations caused by inconsistent histories or data, thereby improving comparability between the 2008 and 2024 methodologies. Another limitation is the narrow scope of diagnoses and impairments assessed. Although this study focuses on common treatment areas, its findings may not be fully generalizable to all conditions. Additionally, the use of only two expert evaluators could limit the applicability of the results, as their assessments may differ from those of less experienced evaluators. Strict adherence to the clinical data presented in the vignettes minimized bias from expertise, ensuring a more objective evaluation, as shown by example 17-3 in the *AMA Guides* Sixth Edition (2008).

Furthermore, while the 2024 *AMA Guides* provide more detailed rating guidance, complex or atypical cases may still require some degree of subjective interpretation when applying impairment criteria. These factors highlight the inherent challenges in impairment evaluation and underscore the need for continued research to validate the application of updated methodologies in diverse clinical scenarios.

While the *AMA Guides* establish impairment values, compensation and disability determinations ultimately rest with the adjudicating jurisdiction. The *AMA Guides* assess impairment, whereas disability decisions extend beyond impairment ratings.

## 5. Conclusions

This comparative analysis evaluated impairment ratings for spine and pelvis conditions using the *AMA Guides* Sixth Editions of 2008 and 2024. The findings support the adoption of the 2024 methodology, as impairment values remained consistent for similar clinical presentations. Key improvements in the 2024 edition include enhanced ease of use, as well as increased consistency, reliability, and reproducibility in both interrater and intrarater evaluations.

The consistent impairment ratings across both editions demonstrate that the 2024 updates maintain the integrity and utility of evaluations while introducing valuable advancements. This balance between continuity and innovation ensures that the *AMA Guides* remain a trusted resource for physical impairment assessments, upholding high standards through thoughtful evolution.

These findings are especially significant for stakeholders—including medical professionals, worker representatives, insurers, legal experts, and policymakers—who rely on the *AMA Guides* for consistent and accurate evaluations. This study offers reassurance that the transition to the 2024 edition will maintain the reliability of impairment assessments while integrating methodological enhancements, alleviating prior concerns about possible disruptions from changes in impairment values. The 2024 methodology features a more structured approach for verifying clinically relevant diagnoses, emphasizing a comprehensive assessment of clinical history, physical examination, and relevant clinical studies. This systematic process enables evaluators to accurately determine the diagnostic row, class, grade, and impairment rating using the enhanced DBI tables, ensuring precise and consistent outcomes.

In summary, the 2024 *AMA Guides* refine established methodologies while retaining core principles from prior editions. The meaningful advancements enrich the evaluation process, reinforcing the *AMA Guides* as the gold standard for impairment assessment and providing stakeholders with confidence in the most current, evidence-based guidelines.

## Figures and Tables

**Table 1:** The 2024 five-step process for spine and pelvis impairment rating.

Step 1. Confirm a Clinically Relevant Diagnosis (DX)
Step 2. Confirm Maximum Medical Improvement (MMI)
Step 3. Identify the Relevant Diagnosis-Based Impairment (DBI) Table
Step 4. Determine the Diagnostic Row, Class, Grade, and Impairment Value
Step 5. Guidelines for Report Documentation

**Table 2:** Comparison of expert evaluators’ impairment ratings, 2008 vs. 2024.

Vignette	WP 2008	WP 2024	WP
17-01	0	0	same
17-02	6	4	less by 2
17-03	10	10	same
17-04	12	14	more by 2
17-05	17	19	more by 2
17-06	28	28	same
17-07	0	0	same
17-08	4	2	less by 2
17-09	12	12	same
17-10	0	0	same
17-11	0	0	same
17-12	1	1	same
17-13	12	12	same
17-14	13	16	more by 3
17-15	19	20	more by 1
17-16	27	29	more by 2
17-17	3	3	same
17-18	6	6	same
17-19	16	16	same
Average	9.79	10.11	
Difference		0.32	

Abbreviation: WP, whole person.

## Data Availability

The original contributions presented in this study are included in the article. Further inquiries can be directed to the corresponding author.

## Abbreviations

The following abbreviations are used in this manuscript:

AMA	American Medical Association
ICF	International Classification of Functioning, Disability, and Health
SIEs	Specific Individual Elements
DBI	Diagnostic-Based Impairment
WPI	Whole person impairment
CCC	Concordance Correlation Coefficient
